# HIGH expression of OSM and IL-6 are associated with decreased breast cancer survival: synergistic induction of IL-6 secretion by OSM and IL-1β

**DOI:** 10.18632/oncotarget.26699

**Published:** 2019-03-12

**Authors:** Ken Tawara, Hannah Scott, Jacqueline Emathinger, Cody Wolf, Dollie LaJoie, Danielle Hedeen, Laura Bond, Paul Montgomery, Cheryl Jorcyk

**Affiliations:** ^1^ Boise State University, Biomolecular Sciences Program, Boise, ID, USA,; ^2^ Boise State University, Department of Biological Sciences, Boise, ID, USA; ^3^ University of Utah, Department of Oncological Sciences, Salt Lake City, UT, USA,; ^4^ Boise State University, Biomolecular Research Center, Boise, ID, USA; ^5^ St. Luke’s Mountain State Tumor Institute, Boise, ID, USA

**Keywords:** oncostatin M, interleukin-6 and oncostatin M, interleukin-1β, breast cancer, metastasis

## Abstract

Chronic inflammation has been recognized as a risk factor for the development and maintenance of malignant disease. Cytokines such as interleukin-6 (IL-6), oncostatin M (OSM), and interleukin-1 beta (IL-1β) promote the development of both acute and chronic inflammation while promoting *in vitro* metrics of breast cancer metastasis. However, anti-IL-6 and anti-IL-1β therapeutics have not yielded significant results against solid tumors in clinical trials. Here we show that these three cytokines are interrelated in expression. Using the Curtis TCGA™ dataset, we have determined that there is a correlation between expression levels of OSM, IL-6, and IL-1β and reduced breast cancer patient survival (*r* = 0.6, *p* = 2.2 x 10^−23^). Importantly, we confirm that OSM induces at least a 4-fold increase in IL-6 production from estrogen receptor-negative (ER−) breast cancer cells in a manner that is dependent on STAT3 signaling. Furthermore, OSM induces STAT3 phosphorylation and IL-1β promotes p65 phosphorylation to synergistically induce IL-6 secretion in ER− MDA-MB-231 and to a lesser extent in ER+ MCF7 human breast cancer cells. Induction may be reduced in the ER+ MCF7 cells due to a previously known suppressive interaction between ER and STAT3. Interestingly, we show in MCF7 cells that ER’s interaction with STAT3 is reduced by 50% through both OSM and IL-1β treatment, suggesting a role for ER in mitigating STAT3-mediated inflammatory cascades. Here, we provide a rationale for a breast cancer treatment regime that simultaneously suppresses multiple targets, as these cytokines possess many overlapping functions that increase metastasis and worsen patient survival.

## INTRODUCTION

Breast cancer-related morbidity and mortality remains one of the top concerns for women worldwide with 266,120 new cases of breast cancer predicted for 2018 [1]. Despite new treatments and extensive preventative screening initiatives, breast cancer incidence remains flat, and there has been little improvement in the survival rate for stage IV metastatic breast cancer over the past decade [2]. One of the contributing factors to this phenomenon may be due to the increasing levels of diabetes and obesity in the developed world which contribute to the development of systemic inflammation [3, 4]. In particular, breast cancer risk factors associated with obesity include metabolic abnormalities and extensive adipose tissue accumulation in the midsection [5]. Strong associations between obesity, cancer, and inflammation have been demonstrated where there has been enhanced breast cancer incidence rates and worsening prognosis with increased obesity [4, 6–8]. Obesity results in elevation of inflammatory cytokines such as interleukin-6 (IL-6), tumor necrosis factor-alpha, (TNFα), and interleukin-1 beta (IL-1β), which have all been linked to the development of breast cancer [9]. IL-6, in particular, has been demonstrated to promote breast tumor cell proliferation and metastatic capacity and to decrease patient survival [10]. Although the importance of IL-6 in cancer disease progression is well documented, anti-IL-6 therapies such as siltuximab have not produced clinically beneficial results for the treatment of solid tumors such as prostate, colorectal, lung, and ovarian cancers, although breast cancer was not yet tested [11, 12]. This lack of effect suggests a potential redundancy, where other pro-inflammatory mediators may also be contributing to breast cancer metastasis and reduced patient survival.

The IL-6 cytokine is a part of the gp130 family of cytokines which include IL-6, oncostatin M (OSM), leukemia inhibitory factor (LIF), IL-11, IL-27, cardiotrophin-1(CT-1), ciliary neurotrophic factor (CNTF), and cardiotrophin-like cytokine factor 1 (CLCF1) [13]. The receptors for each of these cytokines have a shared gp130 subunit and signal a wide range of inflammatory functions driving the pathogenesis of malignancies [13–15]. OSM, in particular, has been shown to induce tumor cell detachment, epithelial-mesenchymal transition (EMT), invasive potential, induction of cancer stem cells, immune evasion, osteolytic bone metastases, and circulating tumor cell numbers [16–23]. OSM functions through binding to the OSM receptor (OSMR), a gp130/ OSMRβ complex, to induce downstream signaling pathways such as signal transducer and activator of transcription 3 (STAT3), mitogen-activated protein kinase (MAPK), and AKT [24–26]. Interestingly, OSM binds to acidic extracellular matrix (ECM) proteins at high concentrations, stays active, and signals downstream pathways in an *in vitro* model of the breast cancer microenvironment [27]. This supports evidence that breast tumors create their own acidic microenvironment and suggest that OSM and other inflammatory factors compound tumor-associated inflammation and lead to increased tumor-cell aggressiveness [28, 29].

Few synergistic interactions between OSM and other pro-inflammatory cytokines have been documented in breast cancer [30, 31]. Synergistic interactions between OSM, interleukin-1 (IL-1α), and IL-1β have been demonstrated in the context of cartilage breakdown in the joint, resulting in an amplified induction of matrix metalloproteinases (MMPs), IL-8, as well as IL-6 expression [32–34]. Additionally, OSM and IL-1β have been shown to synergistically induce vascular endothelial growth factor (VEGF) expression in astroglioma cells [35]. Both IL-1α and IL-1β activate the same IL-1 receptor, (a dimer of IL-1R1 and IL-1RAcP), while IL-1α is a membrane-bound protein and IL-1β is a soluble protein [36]. IL-1β promotes these effects through the activation of NFκB p65 and MAPK-ERK pathways, resulting in the release of cytokines [37–40]. Similar to our published studies, which showed that OSM is important for osteolytic breast cancer metastasis to bone [19], IL-1β also stimulates the development of bone metastases [41]. Unfortunately, anti-IL-1β therapies such as anakinra (Kineret™) have not resulted in improved clinical outcomes for patients with solid tumors, although additional research and clinical trials are currently in progress [42–45]. Furthermore canakinumab, another anti- IL-1β therapeutic agent, had some effect against lung cancer however it had no positive effect on all-cause mortality due to increase in fatal infections [46].

In this study, we investigate the effect of OSM, IL-6, and IL-1β on breast cancer patient survival as well as how these cytokines are interrelated in terms of cell signaling. Using the Curtis TCGA data set [47], we find that high expression of OSM correlates with decreased breast cancer patient survival, similar to previous studies with IL-6 [48]. Previous studies indicated that OSM induces IL-6 in some cell types [49]. Interestingly, OSM induction of IL-6 only occurs in the more aggressive ER− cell lines but not in the ER+ cells lines tested *in vitro*. We also demonstrate that co-treatment of ER− breast cancer cells with both OSM and IL-1β leads to a synergistic increase in IL-6 secretion. These results highlight the complex interactions between OSM, IL-6, and IL-1β, which may render singular anti-cytokine treatments ineffective.

## RESULTS

### Tumor expression of OSM and IL-6 are associated with decreased invasive breast cancer survival and correlate with each other

**Figure 1:**
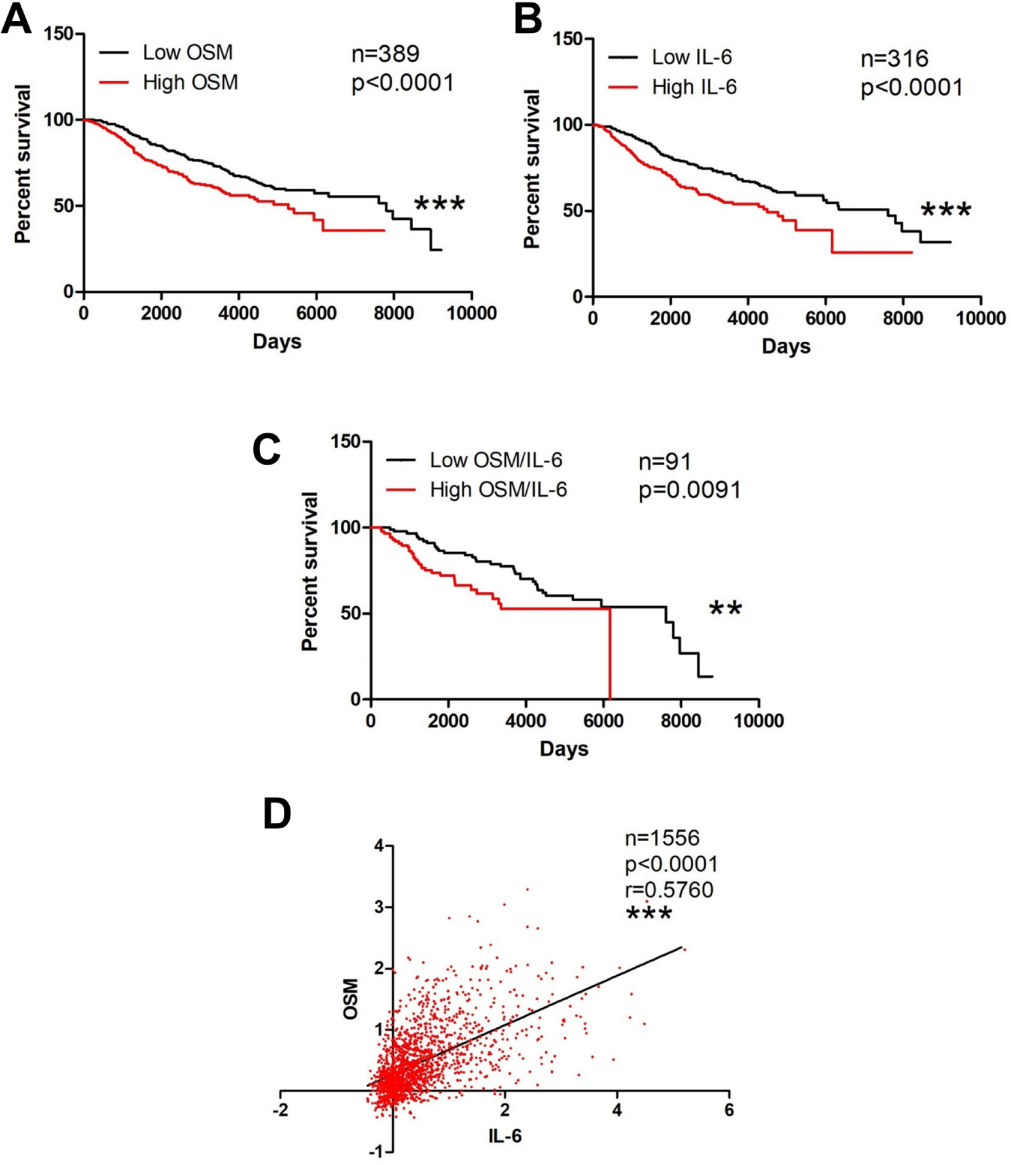
OSM and IL-6 are associated with decreased invasive breast cancer survival (**A**) Kaplan–Meier curves of invasive breast cancer patient samples with high OSM expression levels show significant reduction in survival compared to curves of patients with low OSM expression levels. Log-rank test (*p* < 0.0001) (**B**) This trend is repeated with IL-6. Survival curves of breast cancer patients with high IL-6 expression have reduction in survival compared to patients with low IL-6 expression level Log-rank test (*p* < 0.0001). (**C**) Survival curves of breast cancer patients with high co-expression of OSM and IL-6 also demonstrate a reduction in survival compared to patients with low co-expression of both OSM and IL-6 Log-rank test (*p* = 0.0091). (**D**) Two-way correlation analysis depicts a statistically significant correlation between OSM and IL-6 expression levels in breast cancer patients with a Spearman coefficient of 0.576. (*p* < 0.0001).

Both OSM and IL-6 increase breast cancer metastatic potential *in vitro* as well as promote metastasis *in vivo* [10, 19, 24, 48, 50–54], suggesting that high levels of these cytokines may negatively affect patient survival. In particular, the literature suggests the use of IL-6 as a prognostic marker for breast cancer metastasis and survival [48]. To assess the relevance of tumor tissue expression of OSM and IL-6 in the context of invasive ductal carcinoma (IDC) patient survival, we used the Curtis Breast dataset obtained from Oncomine™ [47]. The upper quartile was delineated as the top 25% of patient expression levels (high expression), while the lower quartile represents the bottom 25% expression (low expression). High tumor tissue expression of OSM (*p* < 0.001, Figure 1A) and IL-6 (*p* < 0.001, Figure 1B) each independently correlated with a significant decrease in invasive breast cancer patient survival. In addition, high co-expression of both OSM and IL-6 were significantly correlated with decreased survival compared to low co-expression of both OSM and IL-6 (*p* = 0.0091, Figure 1C). Further assessment revealed that the breast tumor expression of OSM correlated with the expression of IL-6, with a Spearman coefficient of 0.576 (*p* < 0.0001, Figure 1D). Collectively, these results demonstrate that the breast tumor expression levels of OSM and IL-6 are correlated and that elevated breast cancer tissue levels of these cytokines are associated with decreased survival.

### High serum levels of OSM in breast cancer patients correlate with high IL-6 levels

**Figure 2:**
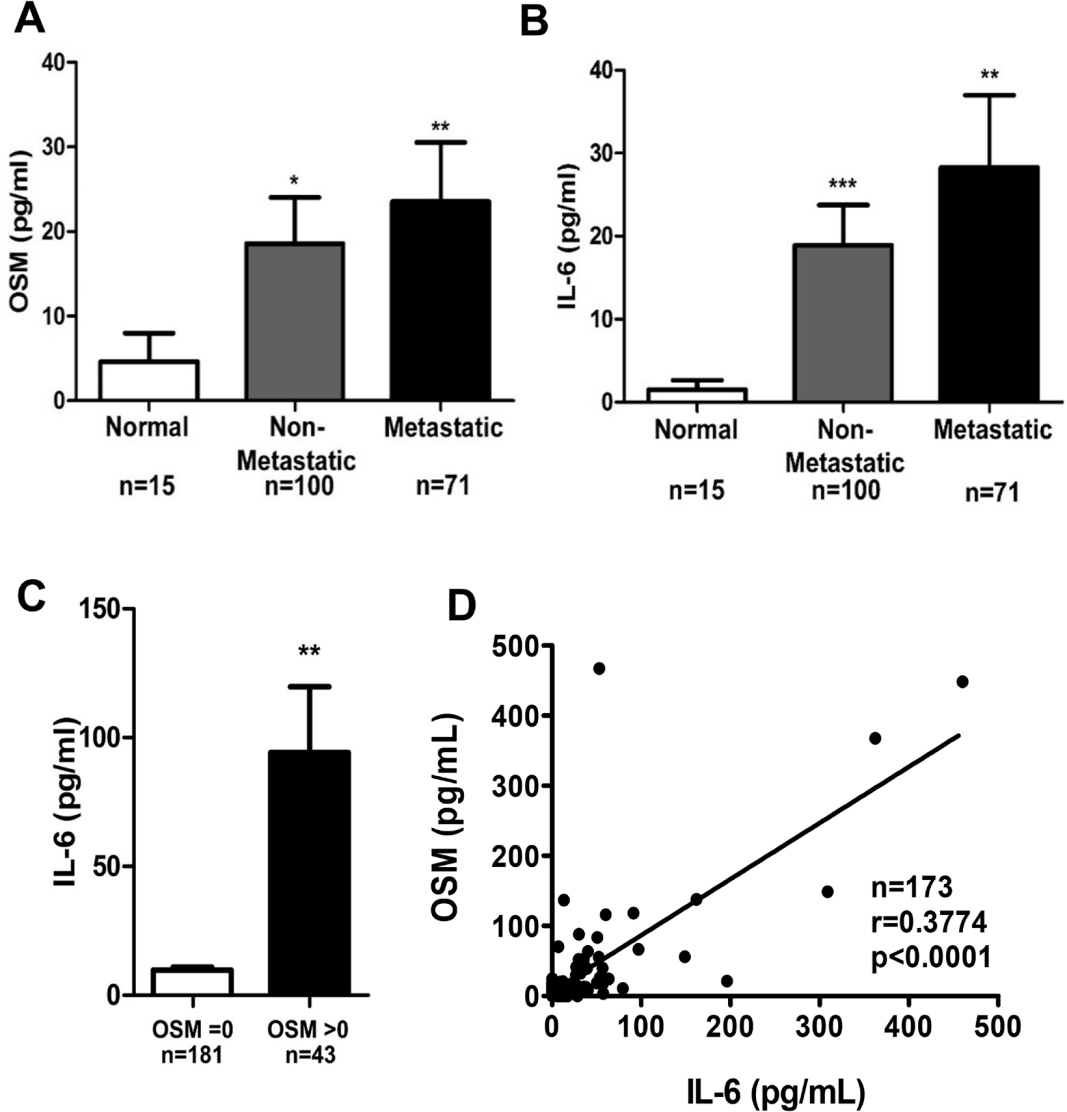
OSM breast cancer patient serum levels correlate with IL-6 levels Serum from breast cancer patients were procured from various sources, and OSM levels (**A**) and IL-6 levels (**B**) were measured by ELISA. When OSM and IL-6 were assessed between normal patients versus patients with non-metastatic or metastatic breast cancer, there was a significant increase in serum OSM and IL-6 levels in both patients with non-metastatic or metastatic breast cancer versus normal patients. (**C**) Patient sera with undetectable OSM levels also have low IL-6 levels (8.5 pg/ml), while patient sera with detectable levels of OSM have high levels of IL-6 (89 pg/ml). (**D**) The serum cytokines concentration data was then assessed for correlation and suggests that higher levels of serum OSM correlates with higher levels of serum IL-6 (Spearman Correlation coefficient = 0.923 (95% CI 0.825, 1.0) *P* < 0.0001). Data expressed as mean ± SEM. and assessed by one-way ANOVA with Tukey’s post-test ^*^p < 0.05, ^**^p < 0.01, ^***^p < 0.001.

Previous studies suggest that elevated expression of growth factors and cytokines in the tumor microenvironment (TME) can result in these proteins leaking into the circulation and becoming detectable in patient serum [55, 56]. To determine whether serum concentrations of OSM and IL-6 also correlate with disease progression and with each other, as seen with breast tumor expression in Figure 1, we assessed serum samples collected from a total of 186 breast cancer patients and healthy individuals by ELISA. First, breast cancer patient serum had significantly higher levels of OSM and IL-6 compared to serum from individuals without malignancies (Figure 2A, 2B). Patients with non-metastatic breast cancer had 3.8-fold higher level of serum OSM compared to normal healthy volunteers, whereas patients with metastatic breast cancer had 4.9-fold higher levels of serum OSM (Figure 2A). Similarly, serum IL-6 levels were 10.5-fold and 15.6-fold higher in non-metastatic and metastatic breast cancer patients, respectively, compared to normal healthy volunteers (Figure 2B). Secondly, serum samples with detectable OSM (> 0 pg/mL) also had significantly higher levels of IL-6, while samples with no OSM contained little to no IL-6 (Figure 2C), suggesting that OSM may cause IL-6 production. Finally, a correlation analysis revealed that serum OSM and IL-6 concentrations were statistically correlated, with a Spearman coefficient of 0.3774 (*p* < 0.0001, Figure 2D). Together, these results clearly demonstrate that breast cancer progression is associated with increased serum levels of both OSM and IL-6 and that the serum concentrations of these two cytokines correlate with each other, similar to the results with tumor expression levels of OSM and IL-6.

### OSM promotes IL-6 secretion in mammary tumors

**Figure 3:**
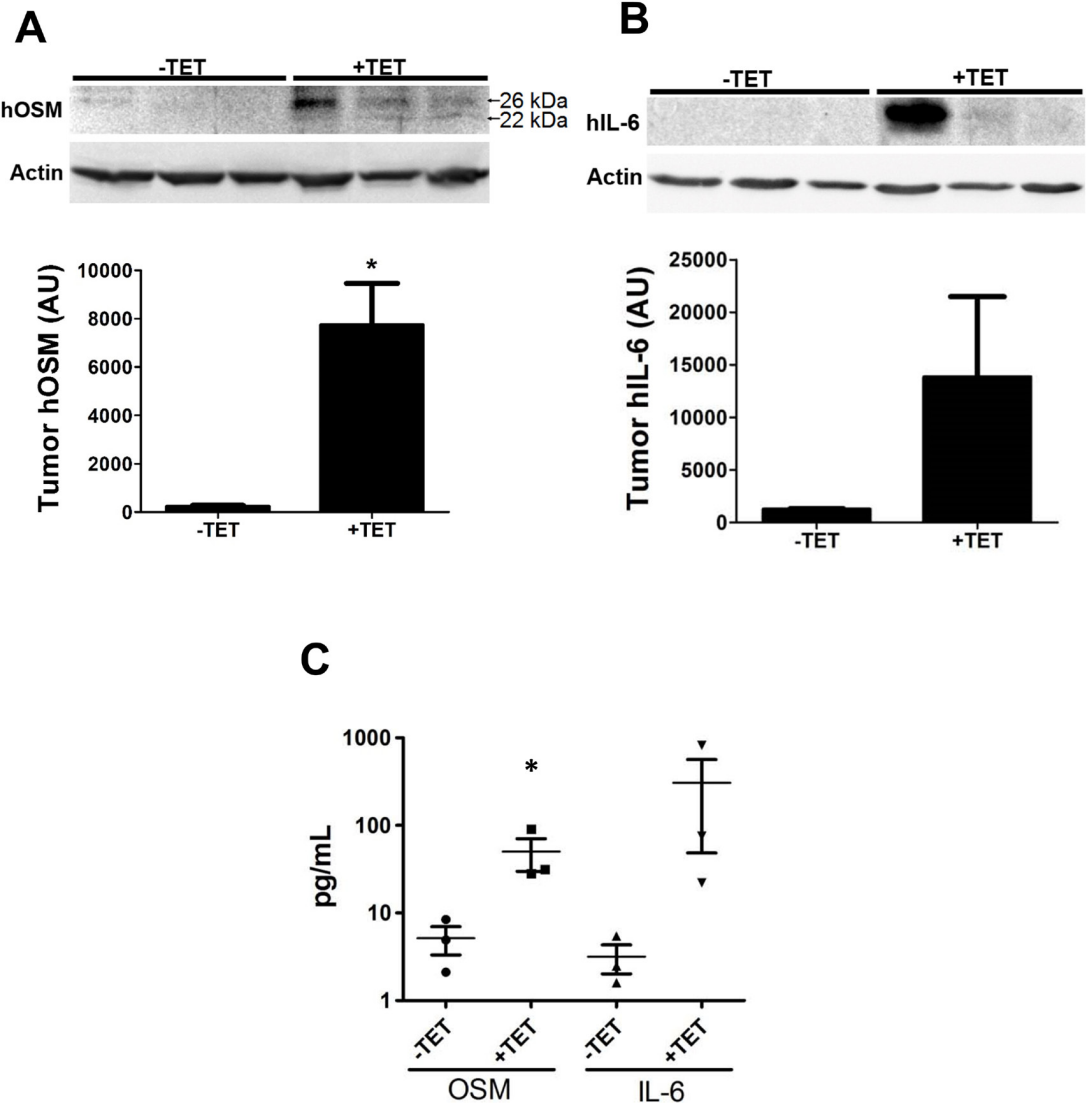
OSM induces IL-6 in an animal model of human breast cancer (**A**) MDA^TO/OSM^ cells were injected orthotopically *in vivo* in athymic nude mice. After the tumors became palpable, the animals were given tetracycline, and after one week, the animals were sacrificed and tumors harvested. Western blot analysis of the tumors indicates that OSM levels increase in response to tetracycline administration (top). Densitometry of western blots indicate a 32-fold increase in tumor OSM in animals given tetracycline compared to animals given control water (bottom). (**B**) IL-6 levels in the tumor, as assessed by western blot, also show similarly elevated levels in the tetracycline-treated animals (10.8-fold). (**C**) Sera collected from the animals were assessed for hOSM and hIL-6 levels by ELISA. Animals given tetracycline have a 9.8-fold increase in mean serum hOSM levels and an 96-fold increase in mean serum hIL-6 levels. Data expressed as mean ± SEM, and significance assessed by two-tailed student’s *t*-test. ^*^p < 0.05.

Our studies with breast cancer patients showed that high serum levels of OSM and IL-6 are correlated, and OSM has been shown to induce IL-6 expression in smooth muscle cells, osteoblasts, and astroglioma cells [49, 57, 58]. This prompted us to investigate whether OSM signals mammary tumor cells to increase IL-6 expression *in vivo*. We utilized an inducible-hOSM MDA-MB-231 orthotopic mouse model of human breast cancer. MDA-MB-231-Luc2 cells were stably transfected with a TET-inducible hOSM expression vector (MDA^TO/OSM^) cells and injected into the 4th mammary fat pad of athymic nude mice. Once the tumors were palpable (∼3–5 mm in diameter), the animals were given drinking water containing tetracycline (+TET; 0.1 mg/mL, *n* = 3) with 2% sucrose or 2% sucrose water alone (−TET, *n* = 3). MDA^TO/OSM^ tumor-bearing animals +TET had a 32-fold higher expression of OSM in their tumors compared to –TET tumors (Figure 3A) and 10.8-fold higher IL-6 expression level (Figure 3B), as measured by western blot analysis. Due to the poor dynamic range of immunoblot imaging, only one mouse in the +TET group appears to have high cytokine levels. A follow up study with cytokines released into circulation, on the other hand, show all three animals in the +TET treated group have elevated OSM and IL-6.

Blood was collected from these mice and hOSM and hIL-6 serum levels were assessed by ELISA. TET-treated mice had higher levels of hOSM (9.8-fold, Figure 3C Left) and IL-6 (96-fold, Figure 3C Right) in their serum, and the concentrations correlated with each other with an r^2^ coefficient of 0.9058 (*p* = 0.0034, Supplementary Figure 1). These findings definitively demonstrate that increased hOSM results in increased hIL-6 expression and secretion in the mammary tumors and serum of MDA^TO/OSM^ mice, which concurs with both our breast cancer patient serum data and the Curtis breast cancer tumor expression data from Oncomine.

### OSM induces human IL-6 secretion in the absence of ER from various cancer cells *in vitro*

**Figure 4:**
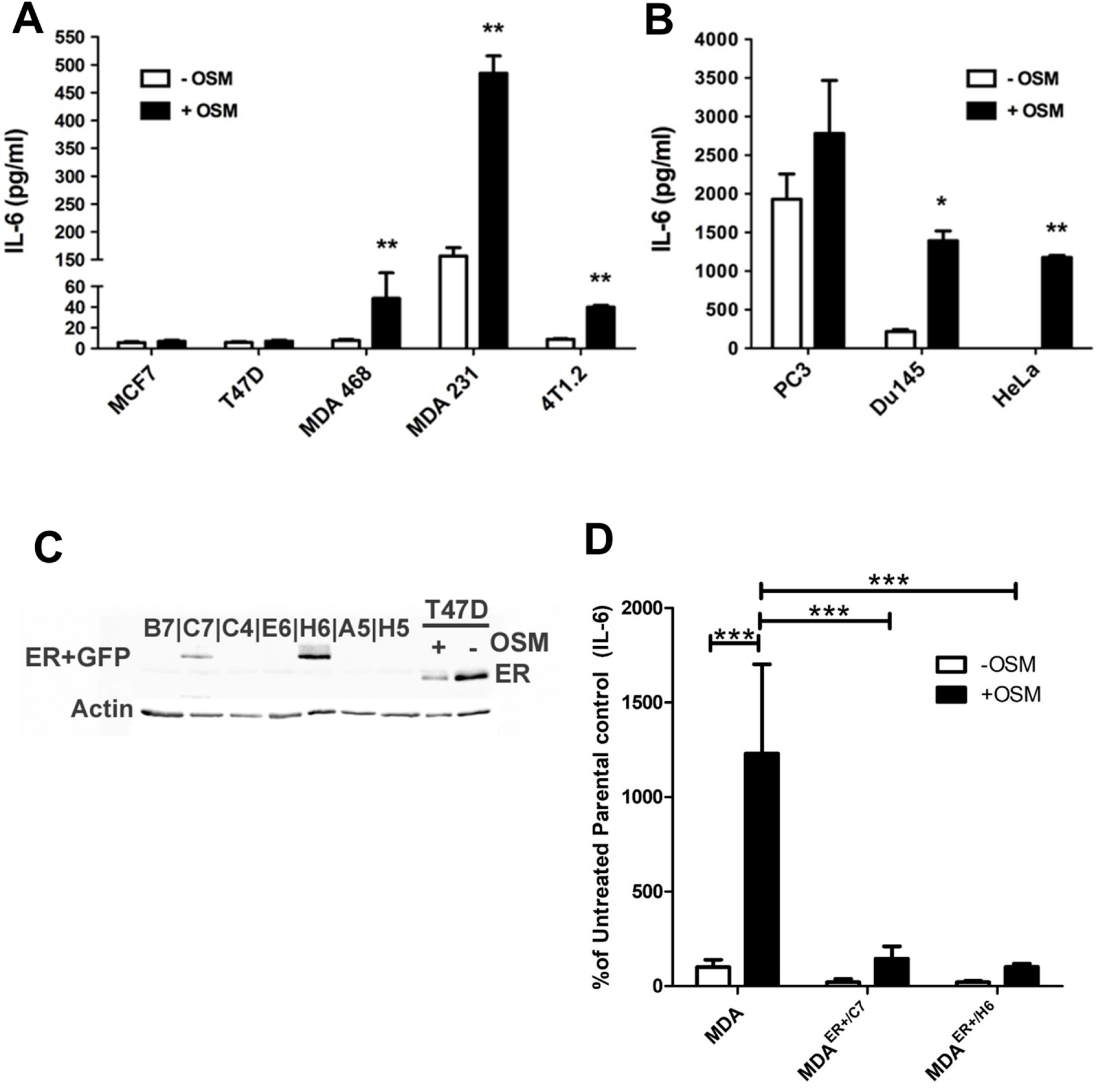
OSM induces IL-6 secretion in an ER-dependent manner (**A**) IL-6 secretion levels were measured by ELISA on CM collected from various OSM-treated cells. ER− MDA-MB-231, MDA-MB-468, and 4T1.2 cells display high (over 4-fold) levels of OSM-induced IL-6 secretion, while ER+ MCF7, and T47D cells do not. (**B**) OSM also induces IL-6 secretion in PC3 and DU145 (prostate) and HeLa (ovarian) ER− non-breast cancer cell types. (**C**) MDA-MB-231 cells were transfected with an ERα expression vector, and the presence of ERα in transfected colonies was determined by western blot. The two ER expressing cell lines are designated as MDA^ER+/C7^ and MDA ^ER+/H6^. (**D**) MDA^ER+/C7^ cells secrete 9-fold less IL-6 and MDA^ER+/H6^ cells secrete 12-fold less IL-6 in response to OSM compared to the parental MDA-MB-231 cells. Data expressed as mean ± SEM and significance assessed by one-way ANOVA with Tukey’s post-test. ^*^p < 0.05, ^**^p < 0.01, ^***^p < 0.001.

Our results indicate that there is a strong correlation between OSM and IL-6 expression and secretion levels in breast cancer. To assess whether OSM induces IL-6 cytokine production in breast cancer cells, various cell lines including two human ER+ cell lines, T47D and MCF7, and three ER− cell lines MDA-MB-468, MDA-MB-231, and 4T1.2 mouse mammary cancer cells were utilized. The cells were treated with human or mouse rOSM for 48 hours, and secreted IL-6 levels in the conditioned media (CM) were assessed by ELISA. Interestingly, OSM did not induce IL-6 secretion in the ER+ MCF7 or T47D cells but did induce IL-6 secretion in the ER− cells (Figure 4A). OSM promoted IL-6 secretion approximately 5-fold in MDA-MB-468 cells, ∼4-fold in MDA-MB-231 cells, and ∼4-fold in 4T1.2 mouse mammary carcinoma cells (Figure 4A). Non-breast cancer, estrogen receptor-negative cell lines were also tested for OSM-induced IL-6 secretion, including PC3 and DU145 human prostate cancer cells, as well as HeLa human cervical carcinoma cells. ER− and androgen receptor-negative (AR-) PC3 cells expressed high levels of IL-6 with or without OSM, while OSM-induced an approximately 6.5-fold increase in IL-6 secretion from ER− AR- DU145 cells, (Figure 4B) and a 30.5-fold in ER− HeLa cells (Figure 4B). Importantly, IL-6 had no effect on OSM secretion in MDA-MB-231, T47D, or MCF7 breast cancer cells (Supplementary Figure 2), suggesting no reciprocal induction of cytokine secretion. These results show that OSM increased IL-6 expression in all aggressive tumor cell lines tested and that this induction may be associated with ER status.

To assess whether the presence of ER suppresses OSM induction of IL-6, we created ER− cells that stably express estrogen receptor. ER− MDA-MB-231 cells were stably transfected with an ERα expression vector (pEGFP-C1). Two independent colonies, MDA^ER+/C7^ and MDA^ER+/H6^, were shown to express ER by western blot analysis (Figure 4C). To assess OSM-induced IL-6 secretion in the new ER+ cells, parental MDA-MB-231, MDA^ER+/C7^, and MDA^ER+/H6^ cells were treated with rhOSM (25 ng/mL) for 48 hours. CM was collected and IL-6 concentrations were analyzed by ELISA. MDA^ER+/C7^ cells exhibited a 7.8-fold decrease, and MDA^ER+/H6^ cells demonstrated a 12.1-fold decrease in the levels of IL-6 produced in response to OSM, as compared to the parental MDA-MB-231 cells (Figure 4D). These results indicate that the ER+ MDA-MB-231 cells have limited OSM-induced IL-6 expression and suggest that ER may play a negative regulatory role in the OSM signaling that leads to IL-6 expression.

### OSM works synergistically with IL-1β to increase IL-6 secretion

**Figure 5:**
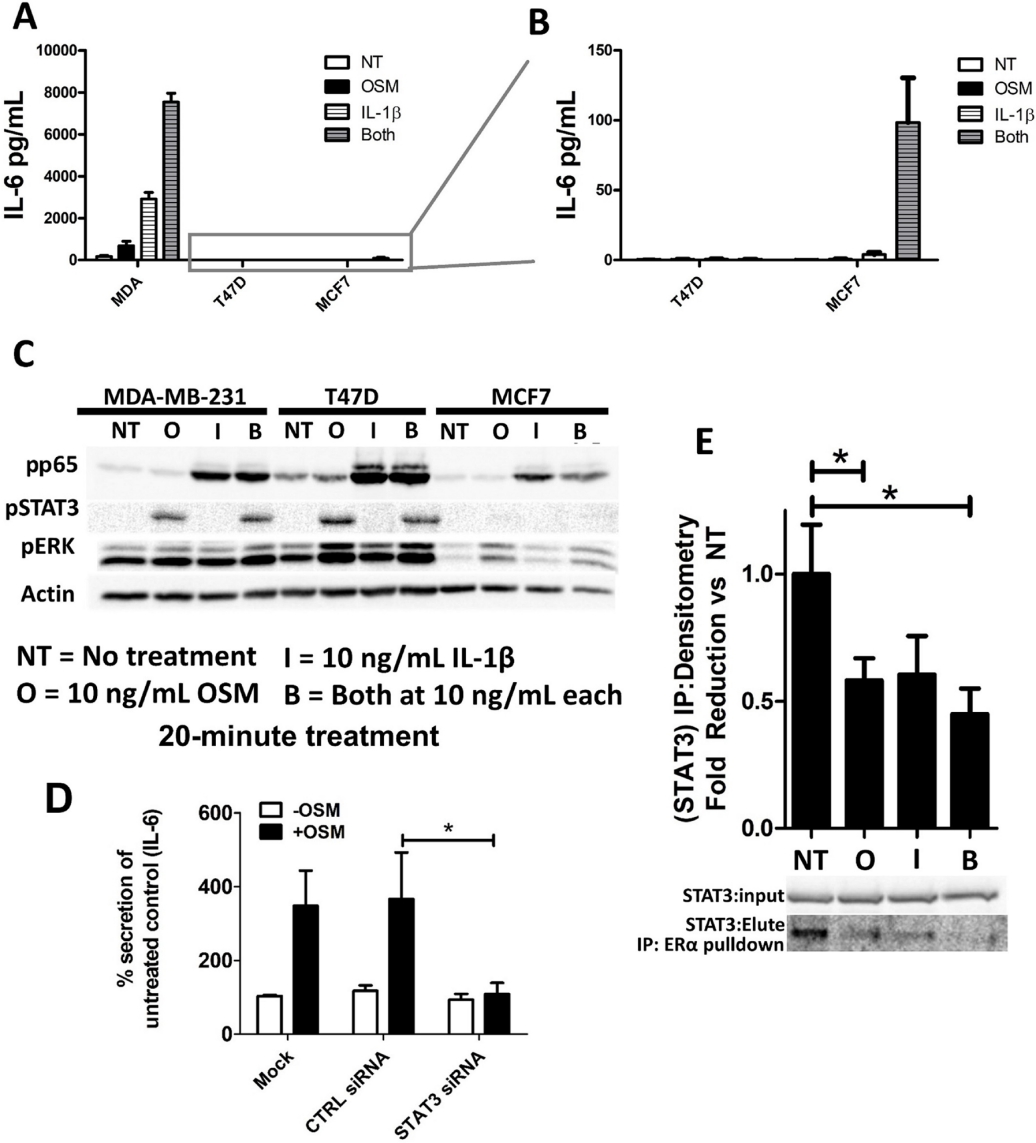
OSM and IL-1β activate separate signaling pathways and synergistically induce IL-6 secretion (**A**) IL-1β alone promotes IL-6 production in MDA-MB-231 and MCF7 cells, while a combination of OSM and IL-1β causes a synergistic response in IL-6 secretion. T47D cells do not produce IL-6 in any of these conditions. (**B**) Reduced IL-6 scale to allow visualization of MCF7 cell-IL-6 induction. (**C**) A 20-minute cytokine treatment with OSM induces the phosphorylation of STAT3 but not p65, while ERK is moderately phosphorylated. MCF7 cells have a weak pSTAT3 induction in response to OSM. A 20-minute cytokine treatment with IL-1β on the other hand induces the phosphorylation of p65 but not STAT3. (**D**) STAT3 siRNA suppressed OSM induction of IL-6 production as assessed by ELISA in MDA-MB-231 cells. Data expressed as mean ± SEM, and significance assessed by one-way ANOVA with Tukey’s post-test. ^*^p < 0.05. (**E**) MCF7 cells were treated with OSM and/or IL-1β for 48 hours, and the cell lysates were run through an immunoprecipitation with an ER pulldown. The eluate was then immunoblotted with the input for STAT3. Lysates collected from MCF7 cells treated with OSM or with both cytokines have significantly reduced ER-STAT3 interaction. Data expressed as mean ± SEM, and significance assessed by one-way ANOVA with Tukey’s post-test. ^*^p < 0.05.

The inflammatory proteins OSM and IL-1β have been demonstrated to have a synergistic effect on IL-6 production in the context of bone and muscle cells [49, 59]. Knowing that inflammation plays a major role in breast cancer pathogenesis, we sought to elucidate whether OSM and IL-1β work together to promote IL-6 secretion in breast cancer. To reduce the probability of saturating the cell’s capacity to produce IL-6 and to better assess a possible synergistic interaction between OSM and IL-1β in breast cancer cells, we decreased the amount of OSM used in the experiments from 25 ng/mL to 10 ng/mL. Treating ER− MDA-MB-231 cells with a combination of OSM and IL-1β (10 ng/mL) for 72 hours resulted in a 44.8-fold increase in IL-6 secretion by ELISA, while OSM alone leads to a 6.4-fold increase and IL-1β alone lead to a 17.3-fold increase compared to untreated cells (Figure 5A). This result suggested that OSM and IL-1β induced IL-6 secretion in a synergistic manner in ER− cells. As demonstrated in Figure 4A, no induction of IL-6 secretion by OSM was seen in either ER+ T47D or MCF7 cells. After adjusting the scale for IL-6 secretion levels, a slight increase in IL-6 secretion by IL-1β was seen in ER+ MCF7 cells, as compared to untreated controls (Figure 5B). MCF7 cells also exhibited a synergistic 24.8-fold increase in IL-6 secretion by treatment with both OSM and IL-1β compared to IL-1β alone (Figure 5B). Although the level of IL-6 production was much lower in the ER+ MCF7 cells compared to the ER− MDA-MB-231 cells, there was a clear indication of a synergistic relationship between OSM and IL-1β in this cell line as well. T47D cells, on the other hand, were unable to produce any IL-6 in response to OSM or IL-1β, even though the cytokine’s activity was confirmed by an increased EMT-like morphology and the formation of invadopodia-like structures (Supplementary Figure 3). These results suggest that OSM and IL-1β may be activating separate pathways to synergistically increase IL-6 secretion. Importantly, these findings also indicate that even when ER+ breast cancer cells, such as T47D cells, are unable to produce IL-6 in response to OSM or IL-1β, they can still undergo invasive characteristics independently of IL-6 [21].

### Early OSM and IL-1β activate STAT3 and p65 signaling pathways to promote IL-6 production

The synergistic upregulation of IL-6 secretion in response to OSM and IL-1β suggests that these cytokines may be using separate pathways to promote IL-6 in breast cancer cells. It is well known that IL-1β utilizes the NFκB pathway to induce IL-6 production [60]. To investigate potential signaling differences between OSM and IL-1β in MDA-MB-231, T47D, and MCF7 cells, the cells were treated with both cytokines (10 ng/mL) for only 20 minutes (Figure 5C), and activated signaling molecules were assessed by western blot analysis. In all breast cancer cell lines tested, OSM specifically induced STAT3 phosphorylation (pSTAT3), while IL-1β induced phosphorylation of p65 (pp65), a subunit of the transcription factor NFκB. In contrast, OSM did not induce pp65 and IL-1β did not induce pSTAT3 (Figure 5C). Additionally, long-term treatment by OSM does not affect total STAT 3 levels [61]. IL-6 has also been known to moderately activate STAT3, [61] but it is unlikely that enough IL-6 accumulated by OSM treatment within 20 minutes to have a significant impact on IL-6 mediated STAT3 phosphorylation.

Further investigation using siRNA (20 nM) against STAT3 showed that siSTAT3 completely abrogated OSM-induced IL-6 secretion in MDA-MB-231 cells as detected by ELISA (Figure 5D), thereby implicating STAT3 as the primary signaling pathway responsible for OSM induction of IL-6 secretion. On the other hand, STAT3 siRNA had no effect on IL-1β-induced IL-6 levels (Supplementary Figure 4). These findings demonstrate a clear role for STAT3 signaling in ER− breast tumor cell OSM-induced IL-6 and confirm that STAT3 is not able to induce IL-6 expression in ER+ T47D or MCF7 cells despite the apparent phosphorylation of STAT3 by OSM.

### ER interaction with STAT3 is suppressed by OSM and IL-1β in MCF7 cells

MDA-MB-231 cells lack ER and subsequently have high levels of IL-6 secretion in response to OSM and IL-1β, while ER+ cells either have no secretion or limited secretion in response to these cytokines. In other studies, ER has been shown to interact directly with various signaling molecules such as p65 and to suppress its downstream signaling despite p65’s apparent activation by protein phosphorylation [62]. To assess whether ER may be binding to intracellular signaling pathway proteins, an immunoprecipitation with ER pull-down was performed on ER+ MCF7 and T47D cell lysates treated with OSM and/or IL-1β for 48 hours. While no interaction between ER and p65, AKT, or ERK was observed (data not shown), a 50% reduction in interaction between ER and STAT3 in response to OSM or IL-1β in MCF7 cells was seen using a STAT3 immunoblot of ER-immunoprecipitation eluates (Figure 5E). With T47D cells, no interaction between ER and any of the signaling proteins was detected (data not shown). This suggests that the interaction of ER with STAT3 may suppress OSM induction of IL-6 in MCF7 cells and that in the ER+ T47D cells, a different mechanism may be operant. Alternatively, there may be cross talk between p65 and STAT3 for downstream signaling [63], and suppression of ER-STAT3 interaction by OSM and IL-1β may be needed for this crosstalk to occur. Our data also show that OSM adversely affects the survival of ER+ patients to a greater extent than the survival of ER− patients (Supplementary Figure 5). This suggests that further ER-specific interactions with OSM signaling may exist, thus requiring future investigation. Collectively, OSM and IL-1β appear to induce IL-6 in a synergistic manner with OSM signaling through STAT3 and IL-1β operating through p65.

### Invasive breast cancer patient stroma expresses high levels of OSM and IL-1β

**Figure 6:**
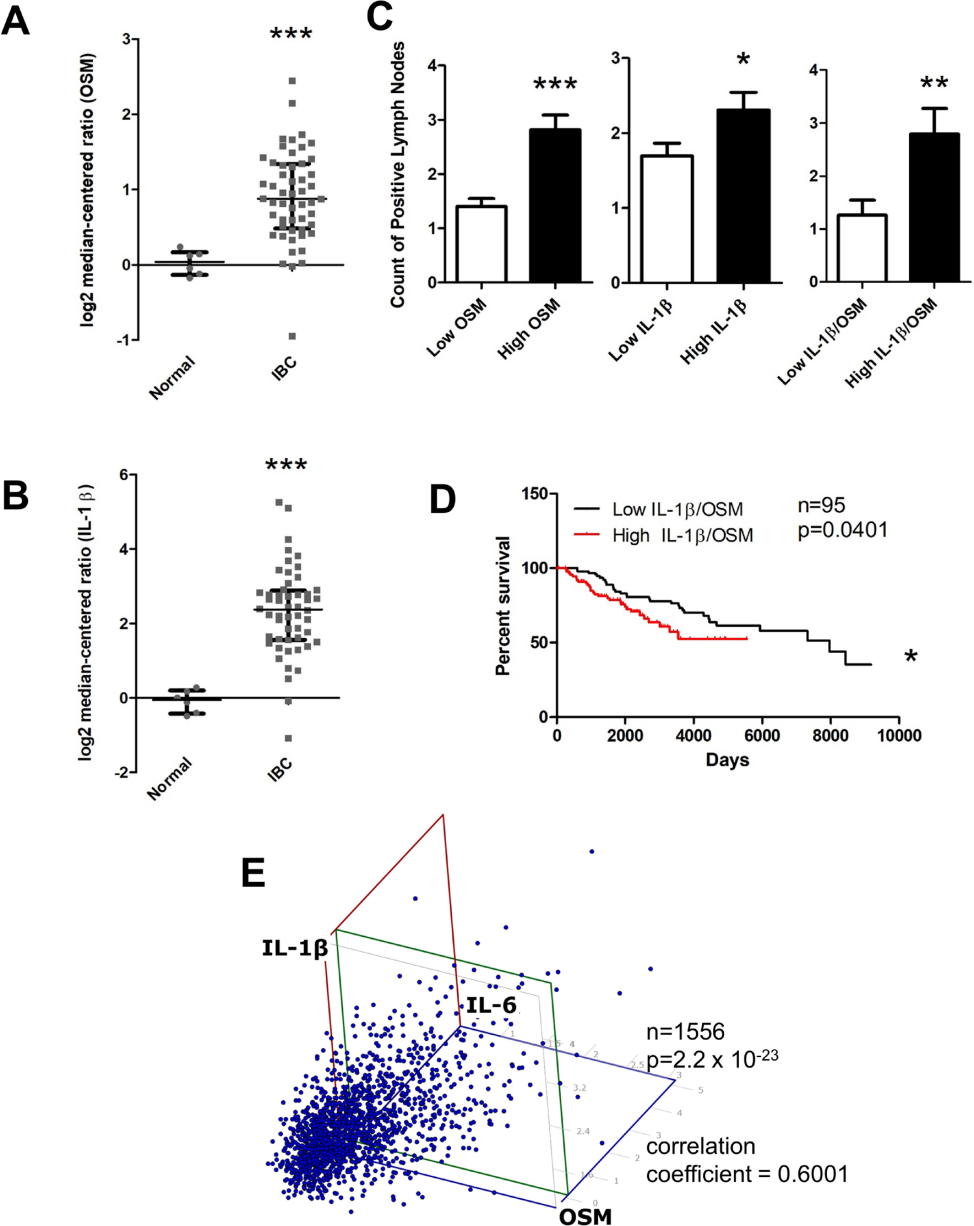
OSM and IL-1β expression is higher in invasive breast cancer compared to normal tissue and correlates with higher lymph node metastasis, decreased survival, and IL-6 levels (**A**) Using the FINAK dataset obtained from Oncomine™ we assessed stromal tissue expression of OSM and IL-1β. Stromal tissue expression of OSM is 5.9-fold higher in invasive breast cancer patients compared to normal patients. (**B**) Similarly, expression of IL-1β is 5.4-fold higher in the stromal tissue of invasive breast cancer patients compared to normal patients. (**C**) Using the Curtis dataset obtained from Oncomine™, we correlated OSM and IL-1β tissue expression levels to the number of lymph node metastases. Patients with high OSM (Left), high IL-1β (Center), and high co-expression of both OSM and IL-1β (Right) have significantly higher number of lymph node metastatic nodules compared to the respective low expression group. (**D**) High co-expression of both OSM and IL-1β leads to a decreased overall patient survival. Log-rank test (*p* = 0.0401). (**E**) Expression of OSM, IL-1β, and IL-6 were analyzed in a three-way correlation analysis with OSM on the x-axis, IL-6 on the y-axis, and IL-1β on the z-axis. There is significant correlation with a coefficient of 0.6001, with a *p*-value of 2.2 × 10^−23^. Bar and scatter plot data expressed as mean ± SEM, and significance assessed by two-tailed student’s *t*-test. ^*^p < 0.05, ^**^p < 0.01, ^***^p < 0.001.

We have demonstrated that OSM and IL-1β act synergistically in the production of IL-6 by breast cancer cells and in the potential exacerbation of inflammatory conditions. It is known that cytokines such as OSM in the stroma of the breast cancer microenvironment play a major role in the progression of metastatic disease [64], and is possible that IL-1β may also have this type of prometastatic effect. To assess the clinical relevance of this synergistic interaction between OSM and IL-1β, we analyzed the stromal expression patterns of OSM and IL-1β in both normal and invasive breast cancer patient data using the Finak Breast Stromal dataset [65] obtained from Oncomine™. Stromal OSM expression was 5.9-fold higher in invasive breast cancer compared to normal breast samples (Figure 6A), and stromal IL-1β was 5.4-fold higher in invasive breast cancer compared to normal samples (Figure 6B). A small but significant increase in stromal OSM (Supplementary Figure 6A) and IL-1β expression (Supplementary Figure 6B) in ER− samples compared to ER+ samples was also seen. Using the Curtis dataset, a significant increase in tumor OSM expression (Supplementary Figure 6C), as well as tumor IL-1β expression (Supplementary Figure 6D), was seen in ER− samples compared to ER+ samples. These data suggest that in breast cancer patients both paracrine and autocrine production of OSM and IL-1β may work in an ER-dependent manner to affect patient survival.

### High tumor OSM and IL-1β levels are associated with increased lymph node metastases and decreased survival

The correlation between tumor OSM and IL-1β expression and metastatic capacity was assessed in the Curtis patient dataset. A 2.1-fold increase in lymph node metastasis seen with high OSM versus low OSM expression for invasive breast cancer (Figure 6C Left). Similarly, lymph node metastasis was higher in IDC patients with high IL-1β expression (Figure 6C Center). Furthermore, when both OSM and IL-1β co-expression was high, there was a 2.3-fold increase in lymph node metastases compared to the low OSM and IL-1β co-expression group (Figure 6C Right). While, IL-1β alone does not appear to affect breast cancer patient survival (Supplementary Figure 7), high co-expression of OSM and IL-1β also led to decreased patient survival (Figure 6D). In addition, OSM expression levels correlated with IL-1β expression level (Supplementary Figure 8A) and IL-1β expression levels correlated with IL-6 expression level (Supplementary Figure 8B). To determine whether all three cytokines were correlated with each other, OSM, IL-6, and IL-1β expression levels were assessed using a least squares multiple correlation analysis (Figure 6E). The three-way correlation coefficient of OSM, IL-6, and IL-1β was 0.6001, with a *p*-value of 2.2 × 10^−23^, indicating a strong correlation between the three cytokines in these IDC tumors. Collectively, these results suggest that OSM, IL-6, and IL-1β are interrelated in breast cancer patient metastasis and survival. Furthermore, these results demonstrate that not only does high expression of each one of these cytokines increase metastasis and decrease survival in breast cancer patients, but OSM and IL-1β also induce the expression of IL-6. This may provide at least in part, an explanation as to why clinical trials using single anti-cytokine therapies thus far have failed for metastatic breast cancer patients.

## DISCUSSION

Previous therapeutic interventions against inflammatory cytokines such as IL-6 and IL-1β have failed in clinical trials despite their well-known role in tumor and metastasis promotion [10, 11, 13]. Nevertheless, with the growing acceptance that chronic tumor inflammation leads to angiogenesis, immunosuppression, proliferation, and metastasis [29], there has been a renewed interest in mitigating inflammation in the tumor microenvironment. In fact, we have recently demonstrated that high tumor expression of OSM or IL-6 along with high VEGF expression is associated with poor survival in HER2- breast cancer patients [61]. Individually, inflammatory cytokines such as OSM, IL-6, and IL-1β have been shown to promote effects associated with metastatic cancer [10, 27, 36, 66]. Our results here suggest that inflammation in the tumor microenvironment may be instigated by multiple cytokines and that the highly-studied IL-6 may only be a limited part of the whole picture. Here we show for the first time the interplay between the three cytokines in breast cancer.

In this study, we reveal a novel finding which demonstrates that high expression of OSM and IL-6 in breast cancer tumors are correlated with reduced breast cancer patient survival. Other studies have linked the OSM receptor to poor prognosis and reduced patient survival is in cervical carcinoma [18, 67] but not to the soluble OSM cytokine. Previous studies have shown that high levels of inflammatory cytokines such as IL-6 increase the risk of breast cancer development and progression [68] and that IL-6 also promotes cancer cell aggressiveness and metastasis to distant organs such as to the bone [10]. While there has been a suggestion that IL-1β may also play a role in breast cancer progression [69, 70], a specific correlation to patient survival has not been previously made.

Our follow up studies using human patient serum demonstrated that high levels of OSM and IL-6 were detected in the serum of breast cancer patients compared to normal healthy volunteers. This was partially recapitulated in an *in vivo* mouse model of breast cancer where mice bearing MDA-MB-231 tumors with a TET-inducible expression of OSM. Not only were the OSM serum levels higher in animals with induced expression of OSM, IL-6 levels were also elevated in the serum. While serum IL-6 levels have previously been demonstrated to be correlated with breast cancer development and reduced survival [71], this is the first study investigating OSM. Our data demonstrate that OSM and IL-6 expression levels correlate in the breast tumor microenvironment, breast cancer patient serum, and in *in vivo* mouse studies.

**Figure 7:**
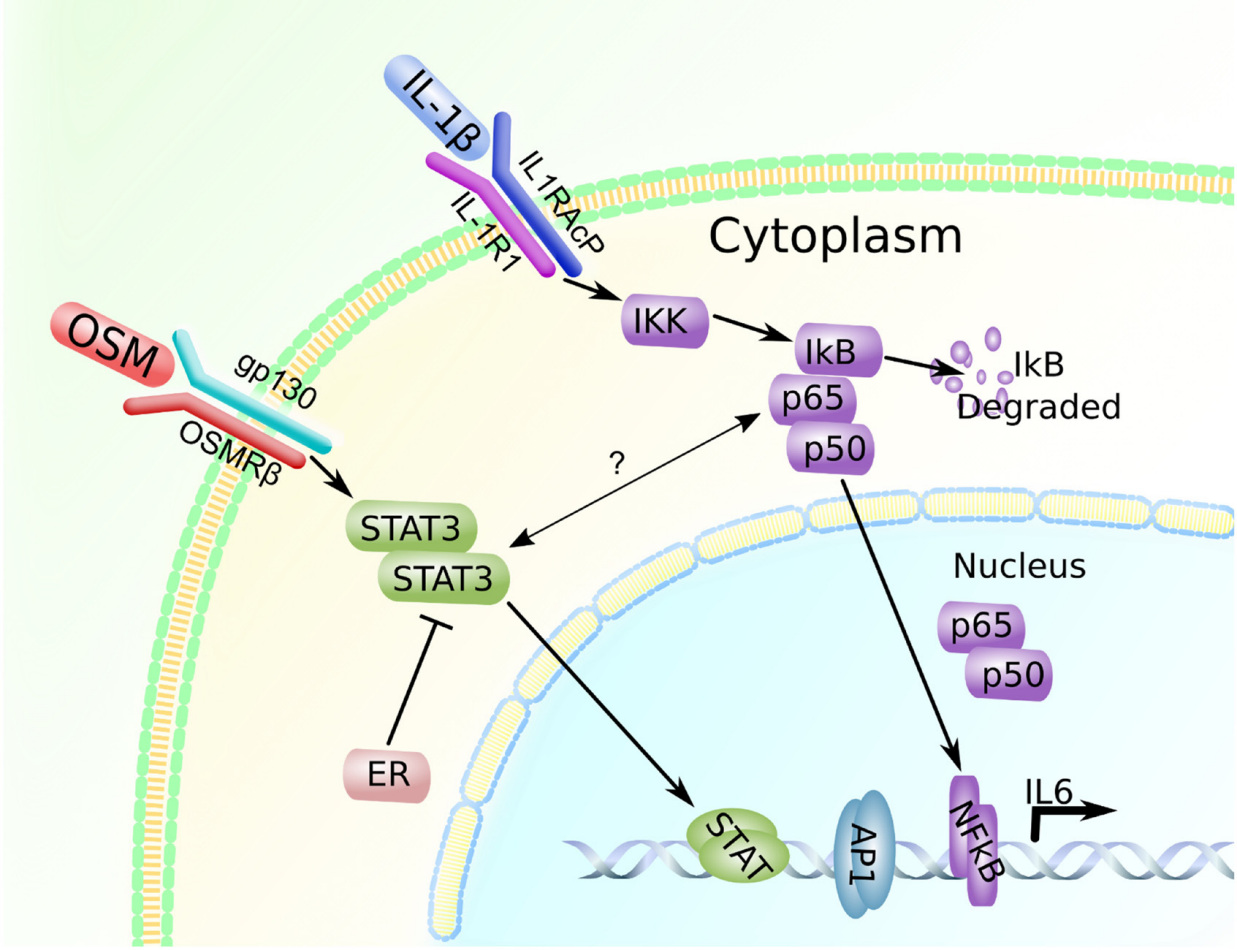
OSM and IL-1β promote IL-6 expression in a breast cancer cell-subtype specific manner OSM signals through the STAT3 pathway and leads to IL-6 induction in ER− cells, while IL-1β induces IL-6 through the p65 pathway. In ER+ cells, ER may be interacting with STAT3 to suppress IL-6 production. There may also be some crosstalk-like interaction between STAT3 and p65, however the exact nature of this interaction is not known [73, 74].

Interestingly, our *in vitro* results demonstrated that OSM induced IL-6 in breast cancer cells in an ER−dependent manner, while OSM did not promote secretion of IL-6 in ER+ cell lines. We showed that suppression of the STAT3 pathway by siRNA reduced OSM-induced IL-6 production to untreated control levels in ER− MDA-MB-231 cells. Constitutive expression of ER in MDA-MB-231 cells also resulted in suppressed OSM-induced IL-6. Previous studies and our data indicate that canonical expression of IL-6 is dependent on the NFκB pathway and that ER suppresses this signaling through inhibition of p65 [62, 72]. However, our immunoprecipitation results suggest that ER may interact with STAT3 to suppress OSM-induced-IL-6 secretion. Taken together, this suggests that OSM induces IL-6 secretion in the MDA-MB-231 cells through the STAT3 pathway and that ER appears to inhibit downstream STAT3 signaling (Figure 7).

Other cytokines such as IL-1β have been known to synergistically interact with OSM in the context of joint damage and synovial fibroblast-mediated inflammation [32, 59]; however, the exact nature of this interaction has not been studied in breast cancer. Our *in vitro* results indicated that OSM works with IL-1β to synergistically increase IL-6 production in MDA-MB-231 cells. On the other hand, ER+ MCF7 cells produced IL-6 in response to IL-1β and OSM co-treatment but not OSM alone, and ER+ T47D cells did not produce IL-6 under any conditions tested. This suggests that despite both of these cell lines being luminal A breast cancer cell subtype (ER+/PR+/HER2-), there exist significant differences in their intracellular signaling mechanisms. We also showed that induction of IL-6 by IL-1β alone or by both OSM and IL-1β was not affected by STAT3 siRNA in either MDA-MB-231 or MCF7 cells. This suggests that OSM and IL-1β work synergistically to increase IL-6 production by activating two separate pathways, STAT3 and NFκB in ER− MDA-MB-231 cells.

Our immunoprecipitation result in MCF7 cells also suggested that treatment of the cells with OSM, IL-1β, or with both cytokines reduced the association of ER with STAT3. Initially, this seems to be an unusual result as IL-1β does not utilize the STAT3 pathway to induce downstream signaling. However, there appears to be some measure of crosstalk reported in the literature between the STAT3 pathway and the p65 pathway, where in some cases the function of the pathway may be interdependent on one another [73]. While the exact nature of the crosstalk mechanism is not well known, it appears that NFκB and STAT3 signaling proteins must be activated and directly bind to each other during signaling [73, 74] (Figure 7). However, if this is the case, STAT3 siRNA should have inhibited IL-1β-mediated IL-6 secretion in MDA-MB-231 and MCF7 cells.

A possible alternative explanation for the lack of any inhibitory activity of STAT3 siRNA on IL-1β-induced IL-6 secretion is that that ER forms an inhibitory complex with STAT3 to suppress IL-6 expression [75]. This is similar to the ER/p65 complex which is known to have a regulatory role in gene expression [75]. This would make suppression of total STAT3 ineffective for reduction of IL-6 expression, as doing so would also render the ER-STAT3 inhibitory complex less effective at regulating IL-6 gene expression. Another possibility is that STAT3 interacts with other STATs in conjunction with ER, such as STAT5, which has some opposing effects against STAT3 signaling in breast cancer cells thus reducing the overall cancer cell aggressiveness [76]. Similarly, STAT1 also appears to have some inhibitory effects against STAT3 signaling [77]. As we have not been able to elucidate the exact mechanism of STAT3/ER interaction and the crosstalk with NFκB, these results necessitate an investigation into pathways that are not necessarily canonically known to be activated by the specific cytokine. Therefore, further investigation into the mechanistic nature of how these signaling pathways interact with each other in the context of breast cancer is needed.

Utilizing the Curtis breast cancer data set, we demonstrated that expression of OSM and IL-1β levels in the breast microenvironment are elevated in breast cancer and that high expression of these cytokines leads to increased metastatic potential and reduced breast cancer patient survival. Previous studies showed similar results where the IL-6 and IL-8 cytokines were associated with increased lymph node metastases and reduced survival [78]. Furthermore, OSM, IL-6, and IL-1β expression levels in breast cancer tissue are all strongly correlated with each other. Other studies have also reported the presence of cytokine co-expression such as tumor necrosis factor alpha (TNFα) expression with IL-6 to have a prognostic significance in breast cancer [79].

Collectively, our study demonstrates that OSM, IL-6, and IL-1β expression levels are correlated with each other and that cytokine signaling differs in an ER subtype-specific manner. OSM and IL-6 have previously been implicated in the production of proangiogenic factors such as VEGF to promote breast cancer progression and reduced patient survival [61]. Together, our results highlight the possible implications of multi-cytokine effects in the tumor microenvironment and have important clinical implications in that singular anti-cytokine therapies may not be sufficient for the successful treatment of metastatic breast cancer. In conclusion, this study substantiates the rationale for a therapeutic design that simultaneously targets multiple cytokines, such as OSM, IL-6, and IL-1β, as these cytokines are strongly correlated with each other in breast cancer.

## MATERIALS AND METHODS

### Oncomine analysis

The Curtis human breast cancer mRNA microarray dataset [47] and the Finak breast cancer stromal gene expression mRNA dataset [65] were obtained from Oncomine™ (Compendia Bioscience, Ann Arbor, MI). For survival analysis, the Curtis dataset was filtered for “Invasive Ductal Carcinoma” and valid “Alive” or “Dead of Disease” status. Patients with the status “Not Dead of Disease” were removed from the study. From the filtered dataset, upper (>75th percentile), and lower (<25th percentile) quartiles of gene expression for OSM, IL-6, and IL-1β were selected for comparison. For multi-gene co-expression analysis, we calculated for patients high in both OSM and IL-6, or OSM and IL-1β. Survival statistical analysis was performed using a log-rank test in GraphPad Prism 5 software.

To analyze gene correlations, the Curtis dataset was subjected to a correlation analysis using GraphPad Prism 5 software and the RealStatistics package on Microsoft Excel. To assess gene expression levels of OSM and IL-1β in stromal tissue, patients in the Finak dataset were separated into normal and breast cancer categories, as well as into ER+ and ER− breast cancer categories. Additionally, the Curtis dataset was also separated into ER+ and ER− breast cancer categories to assess gene expression levels in the tumor cells. Statistical analysis was performed using the 2-tailed student’s *T*-test in GraphPad Prism 5 software.

### Enzyme linked immunosorbent assay

Serum was collected from breast cancer patients of various stages at St. Luke’s Mountain States Tumor Institute (MSTI) and handled in accordance with the St. Luke’s Medical Center (12-0298) and Boise State University (006-MED15-006) Institutional Review Boards (IRB). Additional serum was purchased from Proteogenix (Schiltigheim, France) and Bioreclamations (Baltimore, Maryland). Patients and healthy control subjects with evidence of autoimmune disease or recent infection with bacterial or viral disease were excluded from the study. However, the serum collection was not controlled for either body mass index (BMI) or the presence of metabolic diseases (in the absence of any evidence for auto-inflammatory states), as the effect of these factors appear to be nonspecific on serum cytokine levels. The serum was diluted 1:3 in PBS and used in the DuoSet ELISA for IL-6 (DY206, R&D system) or for OSM (DY295, R&D systems) on Immulon HBX4 ELISA plates (3855, ThermoFisher) in accordance with the manufacturer’s instructions. Data was analyzed using GraphPad Prism 5.0 to determine correlations between OSM-IL-6 serum concentrations.

Cells were incubated with OSM or IL-1β treatments (10 ng/mL) ranging from 48–72 hours and the resultant cytokine levels were assessed. To measure human IL-6 and OSM in conditioned media, the same R&D ELISA kits were utilized according to the manufacturer’s instructions. Conditioned media from MDA-MB-231 cells were diluted 1:10 to 1:20 in order to accurately detect the amount of IL-6 within the range of the standard. Conditioned media was collected from 24-well plates containing 1 × 10^5^ cells at the time of cell plating.

### Cell lines

MDA-MB-231, T47D, MCF7, and MDA-MB-468 human breast cancer cells, PC3 and DU145 human prostate cancer cells, and HeLa human cervical cancer cells were obtained from the American Type Culture Collection (Rockville, MD, USA). All human cell lines were maintained in RPMI 1640 media supplemented with 10% fetal bovine serum and to 100 units/mL of streptomycin and penicillin (Hyclone, Logan, UT, USA). 4T1.2 mouse mammary carcinoma cells were maintained in MEM-alpha media supplemented with 10% fetal bovine serum and to 100 units/mL of streptomycin and penicillin. All cells and experimental incubations were maintained at 37° C, 5% carbon dioxide, and 100% humidity in a water-jacketed cell culture incubator.

### Stable and transient transfections

To generate stably transduced MDA-MB-231 Luc2 D3H2LN cells (Caliper Life Sciences) with inducible expression of OSM, the OSM cDNA (862 bp) (A generous gift from Dr. Atsushi Miyajima, The University of Tokyo) was cloned into the pLenti 6.3/TO/V5-DEST vector (A11144 ThermoFisher). The vector+hOSM was then co-transduced with pLenti3.3/TR (A11144, ThermoFisher) into MDA-MB-231 Luc2 D3H2LN cells using the ViraPower™ II Lentiviral Gateway^®^ Expression System (K367-20, Life technologies) using the manufacturer’s instructions. Stably transduced cells were injected into mice and resultant tumors and animal sera tested for TET induction by western blot and ELISA. The stable TET inducible OSM expressing MDA-MB-231 Luc2 D3H2LN clone has been designated as MDA^TO/OSM^.

To generate stable expression of estrogen receptor alpha (ERα) in ER− MDA-MB-231 cells, an ERα expressing plasmid (Cat# 28230, AddGene) was stably transfected into MDA-MB-231 cells using Lipofectamine LTX (Cat# 15338100, Life Technologies). For control cells, an empty pEGFP-C1 (Cat#6084-1, Clontech) vector was stably transfected into these cells. Cells were transfected at 80% confluency in 96-well plates containing RPMI 1640+10% FBS with 6 µg DNA per well and a Lipofectamine:DNA ratio of 1.35:1. Transfected cells were selected for using G418 at a concentration of 500 μg/mL. Surviving colonies were expanded under antibiotic pressure and their expression of ERα was verified by western blot analysis.

To transiently suppress STAT3, a siRNA pool targeting STAT3 was purchased from Dharmacon (Cat #L-003544-00-0005). 100,000 cells per well were seeded in a 24-well plate, and the siRNA was transfected in accordance to the Fast-Forward protocol as per the technical manual included with the Hyperfect siRNA transfection reagent (Cat# 301705, Qiagen). STAT3 siRNA was used at 25 nM and the cells were transfected for 72 hours before being treated with OSM or IL-1β. Knockdown of STAT3 was assessed by western blot.

### Animal tumor xenograft model

Six-week old female athymic nude mice were purchased from the NCI Animal Production Facility (Fredrick, MD). The MDA^TO/OSM^ cells were grown to 90% confluency and the cells were concentrated to 4.0 × 10^7^ cells/mL in PBS containing 10% RPMI 1640, and 50 uL of the cell suspension was injected into the 4th mammary fat pad. When the tumors became palpable, the animals were given drinking water containing tetracycline in 2% sucrose water for one week with doses ranging from 0 mg/mL, 0.1 mg/mL and 1 mg/mL. Animals were sacrificed and their serum and tumors collected for analysis. All animal experiments were approved by and performed in accordance with the animal guidelines of the Boise Veterans Affairs Medical Center (#JOR0013-1) Institutional Animal Care and Use Committees.

### Immunoblot assay

Cells were plated on 24-well plates at 70-80% confluency and allowed to adhere overnight at 37° C. Cells were treated with cytokines, OSM (CAT#300-10T) and/or IL1-Beta (CAT# 200-01B) (Peprotech), and with inhibitors, the ERK inhibitor PD98059 (CAT# 9900, Cell Signaling) or the p65 inhibitor caffeic acid phenyl ester (CAPE) (CAT# 2743, Tocris). Recombinant hOSM (25 ng/mL) was used to treat all human cell lines, and recombinant mouse OSM (rmOSM; 25 ng/mL) was used for the 4T1.2. Cells were treated for 30 minutes or 72 hours. Conditioned media was collected from the cells treated for 72 hours, and cell lysates were collected from both time points using 1× RIPA buffer containing a protease inhibitor cocktail (Sigma Aldrich, CAT# P8340). Lysates were run on an SDS-PAGE gel and transferred to nitrocellulose immunoblot membranes via semi-dry transfer. Blots were rinsed in ddH2O and allowed to completely dry before being blocked with PBS-T (PBS, pH 7.4; Tween-20,0.05%; 5% non-fat dry milk) for 1 hour. After 3 × 5 min PBS-T washes, primary antibodies (1:1000) suspended in PBS-T complemented with 1% BSA were then applied to the membrane and incubated overnight at 4° C. After another 3 × 5 min PBS-T washes, horseradish peroxidase-conjugated secondary antibodies suspended in PBS-T were then applied to the membrane. Then with a final 5 × 5 min PBS-T wash, the membrane was developed with enhanced chemiluminescence and imaged using Syngene G:BOX imager. All antibodies used for the immunoblots were acquired from Cell Signaling Technologies. STAT3 (CAT#9132), phospho-STAT3 (Y705) (CAT# 9145), Beta-Actin (CAT#3700), NFκB p65 (CAT#8242), phospho-NFκB p65 (CAT#3033), phospho-ERK (CAT# 4370), Anti-rabbit IgG-HRP (CAT# 7076).

### Immunoprecipitation

MCF7 and T47D cells were incubated with 10 ng/mL of OSM and/or IL-1β for 48 hours at a density of 100,000 cells per well in a 24-well plate. Cells were lysed with Cell Signaling PathScan^®^ Lysis buffer (Cat# 7018) using the manufacturer’s instructions. The lysates were then used on a Dynabeads^®^ Protein A Immunoprecipitation Kit (Cat# 10006D, Life Technologies) using ERα IP antibody at 1:50 dilution from Cell Signaling (Cat# D8H8) in accordance with the kit instructions with the following modification. In order to reduce co-elution of the antibody, the antibody was cross-linked using 20 mM dimethyl pimelimidate dihydrochloride (Cat# 21666, Pierce) in 0.2 M triethanolamine at a pH of 8.2. The antibody-bead complex was cross-linked for 30 minutes, and the reaction was stopped by resuspending the beads for 15 minutes in 50 mM pH7.5 Tris. The beads were then used in the rest of the immunoprecipitation protocol following a 3× PBS-0.05% Tween-20 wash.

### Statistical analysis

All the statistical analyses were performed using GraphPad Prism version 5 software or the RealStatistics™ package for Microsoft Excel. To compare multiple groups, one- or two-way ANOVA was performed using Tukey’s post-test where appropriate. Comparisons between two groups were assessed by unpaired two-tailed student’s *t*-test. Correlations were assessed using the Spearman nonparametric correlation analysis. Survival data was assessed using the Log-rank test. Statistical significance was assigned to experimental *p* values that were less than 0.05. Error bars represent the standard error of the mean unless otherwise specified, and all experiments were performed at least three times.

## SUPPLEMENTARY MATERIALS FIGURES


