# Supplementary material for: HIGH expression of OSM and IL-6 are associated with decreased breast cancer survival: synergistic induction of IL-6 secretion by OSM and IL-1β
Source: Oncotarget. 2019 Mar 12;10(21):2068–85. doi: 10.18632/oncotarget.26699 (PMC6459341; doi:10.18632/oncotarget.26699)
Supplement: Supplementary file 1 [file oncotarget-10-2068-s001.pdf]

HIGH expression of OSM and IL-6 are associated with decreased breast cancer survival: synergistic induction of IL-6 secretion by OSM and IL-1β

SUPPLEMENTARY MATERIALS

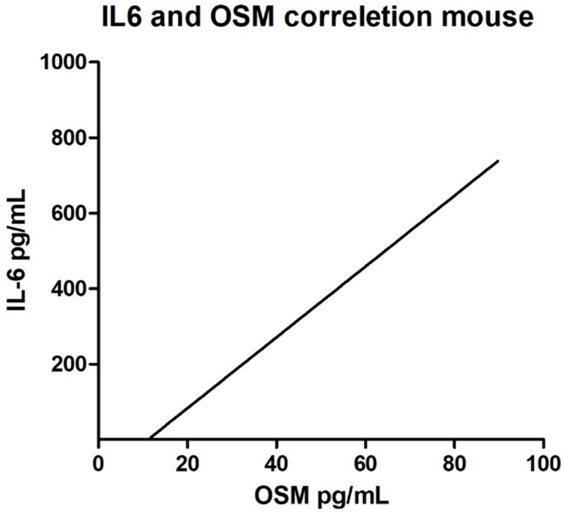

Spearman coefficient of 0.9058 **p=0.0034**

Supplementary Figure 1: OSM and IL-6 serum concentrations are correlated *in vivo*. OSM and IL-6 serum concentrations in the MDA$^{TO/OSM}$ mouse model were assessed by ELISA, and the resultant numbers were analyzed using a correlation analysis. OSM and IL-6 levels correlate with a spearman correlation coefficient of 0.9058 and a *p*-value of 0.0034.

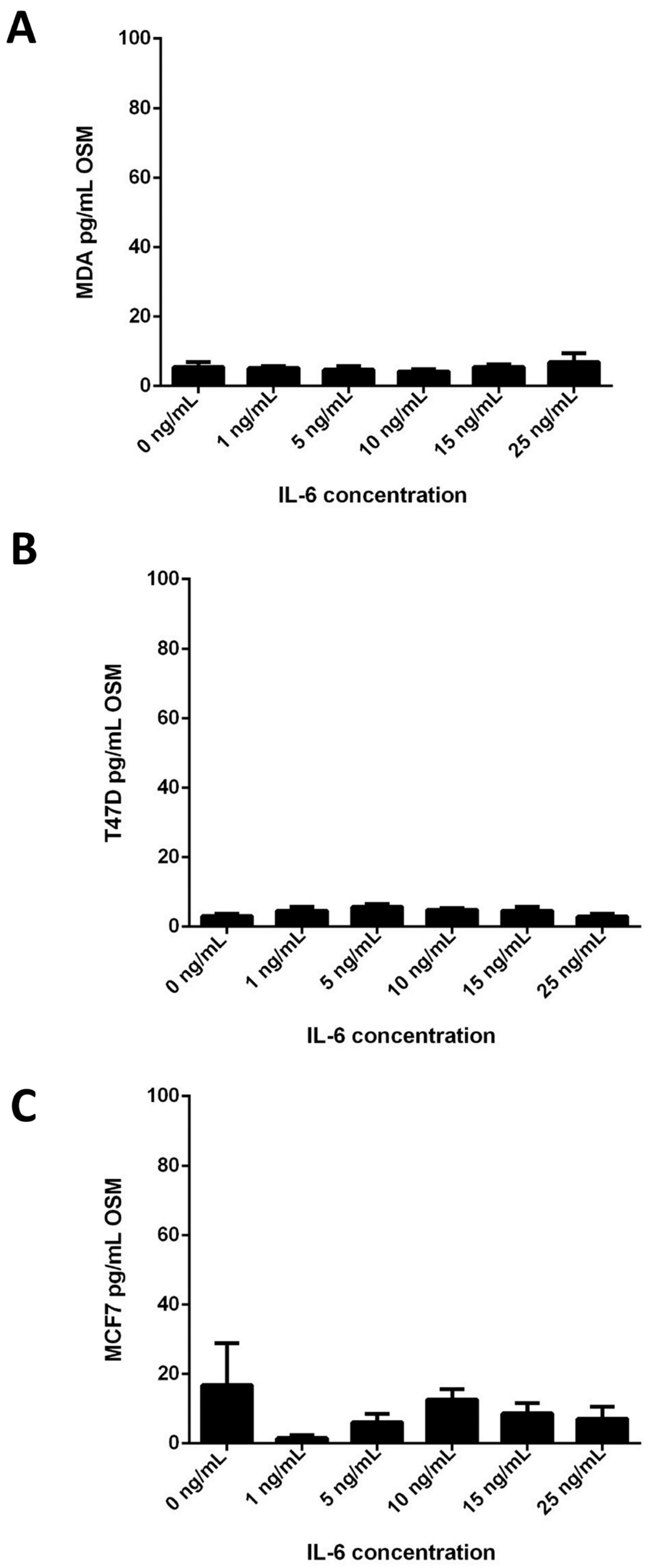

Supplementary Figure 2: IL-6 does not promote OSM secretion in human breast cancer cells. (**A**) MDA-MB-231, (**B**) T47D, and (**C**) MCF7 human breast cancer cells were treated with IL-6, and secreted OSM levels in the conditioned media were assessed by ELISA. IL-6 did not promote OSM secretion in any of the cell lines tested.

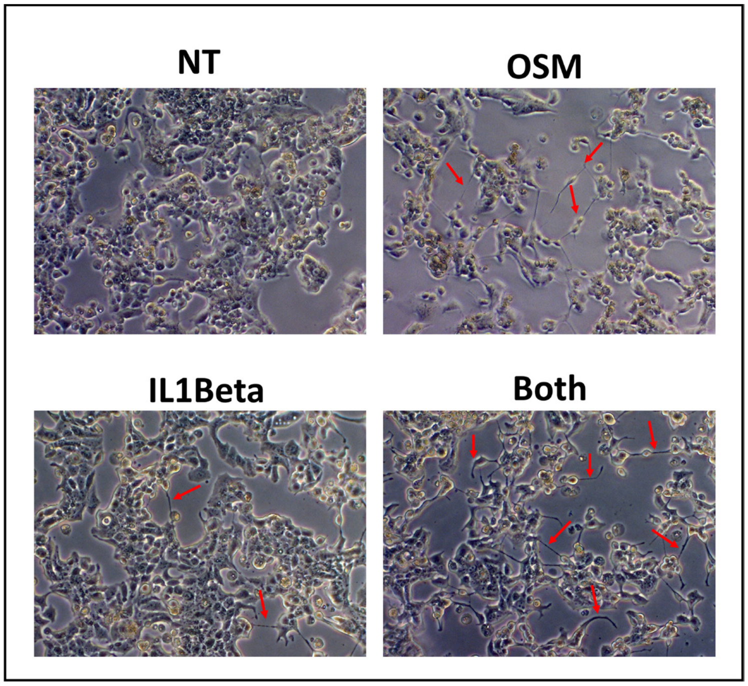

Supplementary Figure 3: OSM and IL-1β promote EMT-like morphology in T47D cells. T47D cells do not secrete IL-6 under any treatment conditions. To assess whether the cytokines have an effect on T47D cells, they were treated with 10 ng/mL of OSM and/or IL-1β for 72 hours, and the cell morphology was assessed by phase contrast microscopy. Treatment of the cells with OSM and/or IL-1β promotes an EMT-like cell morphology, with the cells becoming more mesenchymal and spreading apart from each other. Red arrows indicate areas with invadopodia-like structure formation. These results indicate that OSM and IL-1β signal T47D cells to alter their morphology, even though these cytokines do not result in an increase in IL-6 secretion.

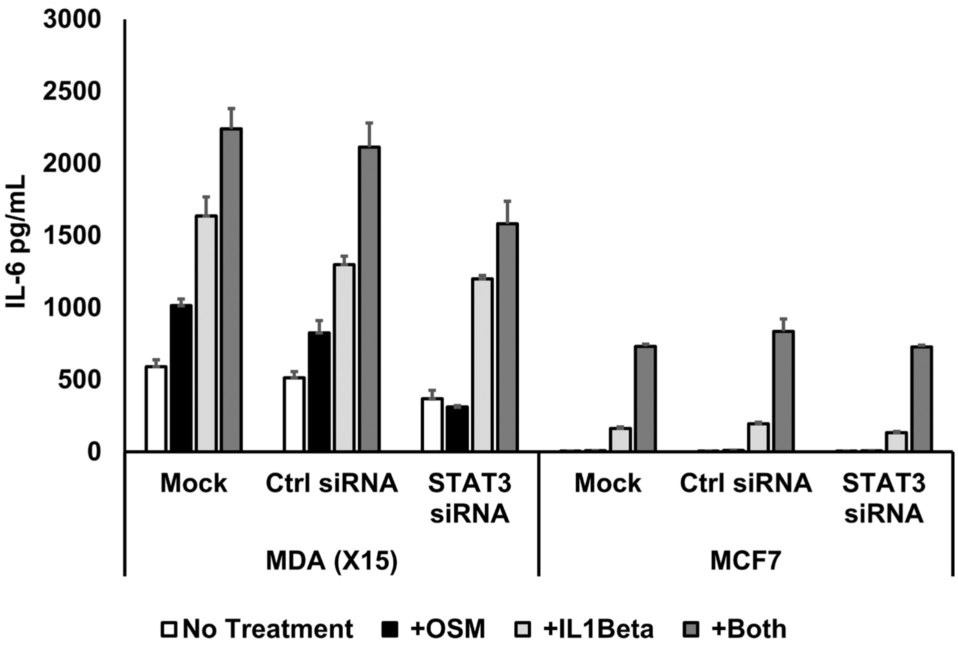

Supplementary Figure 4: STAT3 siRNA does not affect IL-1β-induced IL-6 secretion. MDA-MB-231 and MCF7 cells were treated with 25 nM of STAT3 siRNA with or without 10 ng/mL of OSM and/or IL-1β for 72 hours. Although the STAT3 siRNA suppresses OSM-induced IL-6 secretion, it has no effect on IL-1β-induced IL-6 secretion.

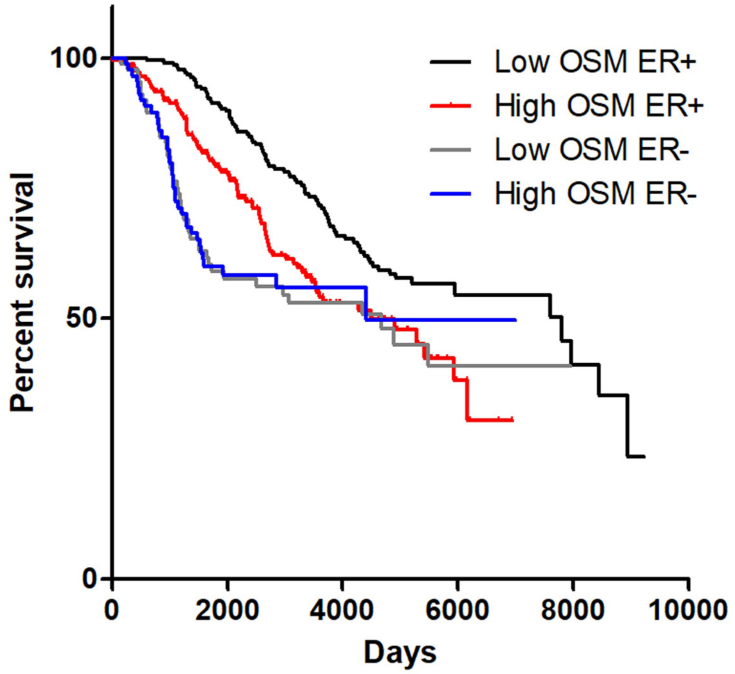

Supplementary Figure 5: OSM is associated with poor survival in ER+ breast cancer patients. ER+ breast cancer patients with high levels of serum OSM have lower survival rates than ER+ patients with low levels of OSM. However, ER− breast cancer patients overall have the lowest survival rates.

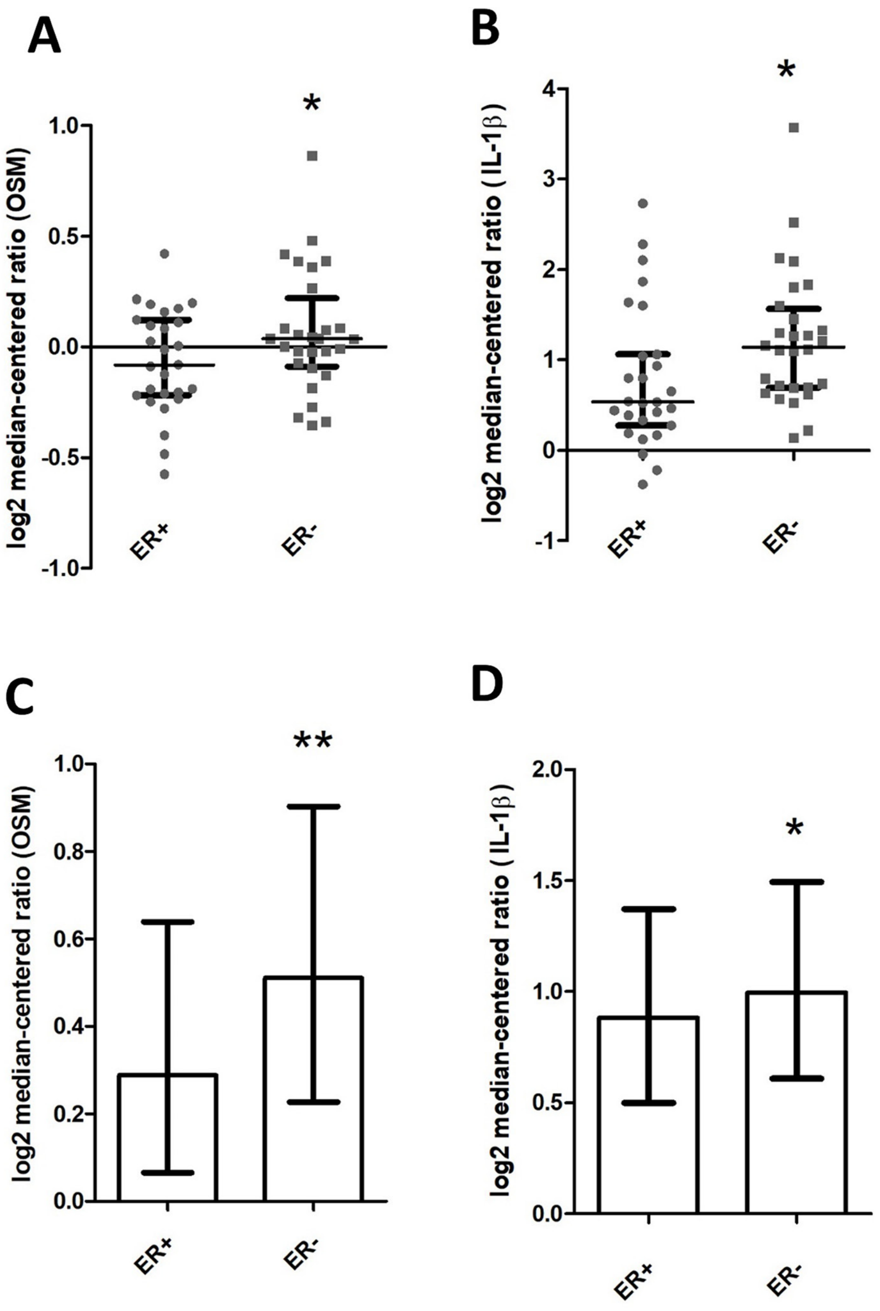

Supplementary Figure 6: Stromal and tumor tissue expression of both OSM and IL-1β are higher in ER− patients. (**A**) Stromal OSM and (**B**) IL-1β expression is significantly higher in patients with ER− breast cancer compared to ER+ breast cancer, as assessed using the Finak Oncomine™ dataset. Additionally, using the Curtis Oncomine™ dataset, tumor tissue expression of OSM (**C**) and IL-1β (**D**) is also significantly higher in ER− breast cancer patients compared to patients with ER+ breast cancer. Data expressed as $\log_2$ median-centered ratio ± SD and significance assessed by two-tailed student's *t*-test. $^{*}p < 0.05$, $^{**}p < 0.01$, $^{***}p < 0.001$.

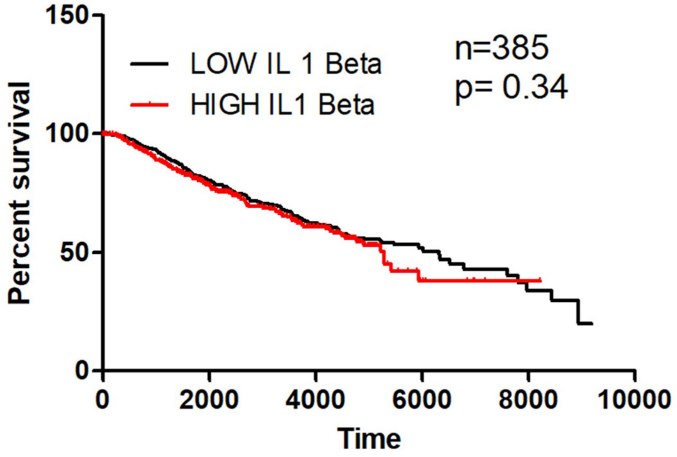

Supplementary Figure 7: IL-1β expression levels alone are not associated with breast cancer patient survival. The Curtis breast cancer dataset was assessed for IL-1β expression and patient survival. No significant difference in survival was detected between patients with low versus high IL-1β expression. Log-rank test ($p = 0.34$).

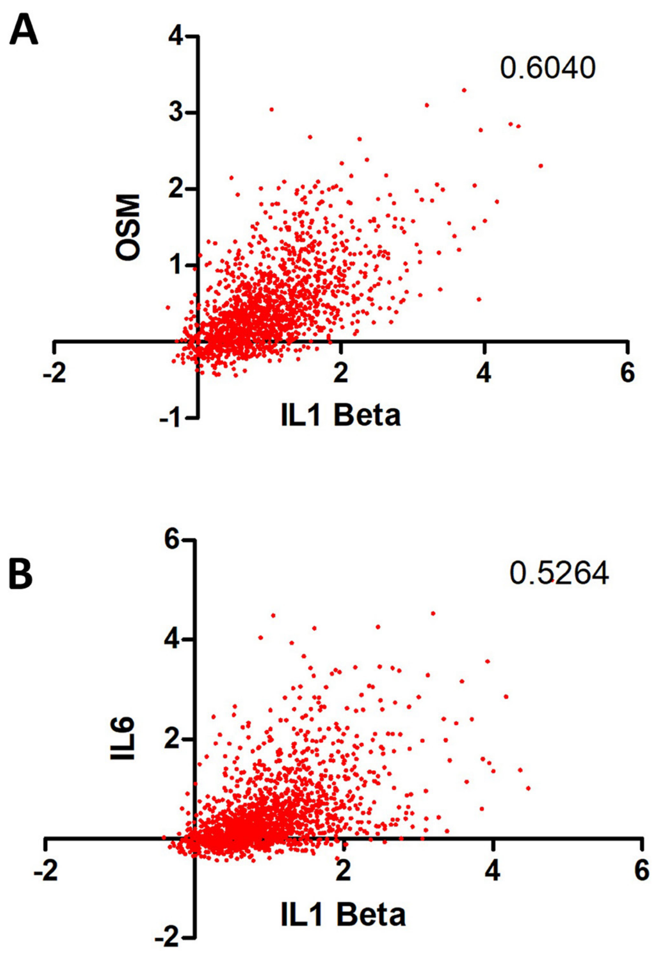

Supplementary Figure 8: IL-1β mRNA expression levels correlate with both OSM and IL-6 in breast cancer patients. The Curtis breast cancer dataset was assessed for OSM, IL-6, and IL-1β tumor mRNA expression levels. (**A**) OSM and IL-1β expression levels correlate with each other with a spearman coefficient of 0.604 with a *p* value of less than 0.0001. (**B**) IL-6 and IL-1β expression levels correlate with each other with a spearman coefficient of 0.526 and a *p* value of less than 0.0001. Correlation coefficients were assessed using the Spearman correlation analysis.
